# Ecological Niche Differentiation and Response to Climate Change of the African Endemic Family Myrothamnaceae

**DOI:** 10.3390/plants13111544

**Published:** 2024-06-03

**Authors:** Qisong Wan, Shenglan Du, Yu Chen, Feng Li, Radwa Salah, Maxwell Njoroge Njenga, Jitao Li, Shengwei Wang

**Affiliations:** 1 Hubei Key Laboratory of Biologic Resources Protection and Utilization, Hubei Minzu University, Enshi 445000, China; qisongwan@outlook.com (Q.W.); chenyu22824@outlook.com (Y.C.); 2 Key Laboratory of Plant Germplasm Enhancement and Specialty Agriculture, Wuhan Botanical Garden, Chinese Academy of Sciences, Wuhan 430074, China; dusl2021@outlook.com (S.D.); lifeng211@mails.ucas.ac.cn (F.L.); radwasalah979@gmail.com (R.S.); nmaxwell@jkuat.ac.ke (M.N.N.); 3 Center of Conservation Biology, Core Botanical Gardens, Chinese Academy of Sciences, Wuhan 430074, China; 4 School of Ecology and Environment, Tibet University, Lhasa 850000, China; 5 University of Chinese Academy of Sciences, Beijing 100049, China; 6 Sino-Africa Joint Research Center, Chinese Academy of Sciences, Wuhan 430074, China

**Keywords:** ecological niche, adaptive evolution, climate change, species distribution modeling, Myrothamnaceae

## Abstract

Studying the ecological niches of species and their responses to climate change can provide better conservation strategies for these species. Myrothamnaceae is endemic to Africa, comprising only two species that belong to *Myrothamnus* (*M. flabellifolius* and *M. moschatus*). These closely related species exhibit allopatric distributions, positioning them as ideal materials for studying the species ecological adaptation. This study explores the ecological niche differentiation between *M. flabellifolius* and *M. moschatus* and their response capabilities to future climate change. The results indicate that *M. flabellifolius* and *M. moschatus* have undergone niche differentiation. The main drivers of niche differences are the minimum temperature of the coldest month (Bio6) for *M. flabellifolius*, precipitation of the driest month (Bio14), and precipitation of the coldest quarter (Bio19) for *M. moschatus*. *M. flabellifolius* demonstrated a stronger adaptation to environments characterized by lower precipitation, relatively lower temperatures, and greater annual temperature variations compared to *M. moschatus*. Under future climate scenarios (SSP5-8.5, 2081–2100 years), the results show that approximately 85% of the total suitable habitat for *M. flabellifolius* will be lost, with an 85% reduction in high-suitability areas and almost complete loss of the original mid-low suitability areas. Concurrently, about 29% of the total suitable habitat for *M. moschatus* will be lost, with a 34% reduction in high suitability areas and roughly 60% of the original mid-low suitability areas becoming unsuitable. This suggests that *M. flabellifolius* will face greater threats under future climate change. This study contributes novel insight into niche differentiation in Myrothamnaceae and provides useful information for the conservation of this distinctive African lineage.

## 1. Introduction

Adaptive evolution is a fundamental biological process where populations evolve adaptive traits over generations, driven by natural selection and other mechanisms like mutation and gene flow [1]. This process not only enhances species adaptability and biodiversity but also helps to drive ecological speciation through their niches [2]. The ecological species concept highlights the role of these niches in understanding how species adapt to environmental pressures, thereby aiding the study of speciation and biodiversity conservation [3]. Understanding the ecological niches and adaptability of species helps us to elucidate the mechanisms of speciation and the maintenance of diversity. Meanwhile, it unveils the mechanisms and patterns of adaptive evolution in species [4,5].

The theory of species adaptive evolution emphasizes the importance of adaptation not only to static environments but also to changing environments. Climate is one of the most important factors in the biological environment [6]. In response to climate change, organisms often gain opportunities for individual and species survival by altering certain adaptive traits [7]. For example, some animals alter their behaviors, such as hibernation, reproduction, and migration [8,9]. Similarly, plants can gradually adapt to climate change by adjusting their phenology, metabolism, morphological features, and other functional traits [10,11]. These adaptive changes in organisms are accompanied by changes in their ecological niches, which are highly unfavorable for species with strong niche conservatism [12]. Climate change directly threatens the survival of these species and accelerates the extinction of some endangered species [13,14]. A previous study indicated that approximately one-third of vascular plants in Africa are threatened with extinction due to climate change [15]. Therefore, exploring the adaptive capacity of different species to climate change and enhancing our understanding of the relationship between climate change and species ecological niche differentiation will help predict the responses of species to climate change and implement effective conservation and management measures. This is essential for the protection of Earth ecosystems and biodiversity and for promoting harmonious development between humans and nature.

The African endemic family Myrothamnaceae, belonging to the order Gunnerales, consists of a single genus, *Myrothamnus*, which only comprises *M. flabellifolius* Welw. and *M. moschatus* Baill [16,17]. Myrothamnaceae is a sister family to Gunneraceae, with the divergence dated to approximately 80 million years ago (MYA) [16,17,18]. *M. flabellifolius* and *M. moschatus* diverged about 7 MYA [18]. These two species display significant geographic separation in their distribution patterns. *M. flabellifolius* is distributed in southern Africa, while *M. moschatus* is only found in Madagascar [16]. Additionally, they demonstrate distinct ecological adaptabilities and functional trait differentiation [16,17]. *M. flabellifolius* and *M. moschatus* are known as resurrection plants due to their desiccation tolerance [16,17]. They can enter a state of dehydration dormancy during water scarcity and swiftly revive once rehydrated [19]. *M. flabellifolius* features fan-shaped leaves and possesses hydathodes, while *M. moschatus* has smaller, narrower, lanceolate leaves without hydathodes [16]. Moreover, the stomata on *M. moschatus* leaves are sunken, contrasting with the non-sunken stomata of *M. flabellifolius* [16]. Furthermore, the xylem vessels of *M. flabellifolius* include annular structures, which are absent in *M. moschatus* [16]. The variation in plant traits often indicates different adaptations to the external environment, corresponding to niche differentiation [20]. However, whether niche differentiation occurs in Myrothamnaceae and what factors underly this differentiation is unclear.

Severe climate change has significant impacts on many xerophytic plants [21]. Myrothamnaceae plays a significant role in dry ecosystems because it acts as a pioneer species in bare rock habitats and facilitates the establishment of other plants [17]. Research indicates that most regions in Africa are currently experiencing rising temperatures, reduced rainfall, and extreme weather events [22]. However, Myrothamnaceae has not yet been included in the IUCN list of endangered plants, and the impact of climate change on it remains unknown [23]. This study focuses on the Myrothamnaceae to explore its ecological niche differentiation and its response capabilities to future climate change (SSP5-8.5, 2081–2100 years). This research aimed to enhance our understanding of the ecological mechanisms of species adaptive evolution and provide scientific insights for the conservation of these species.

## 2. Results

### 2.1. The Distribution Pattern of Myrothamnaceae

**Figure 1:**
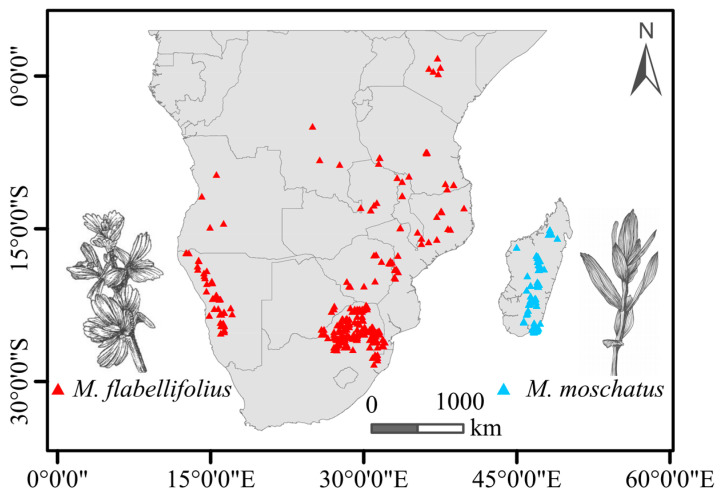
Summary of present-day known distribution (points) of the two Myrothamnaceae species in Africa.

Based on information obtained from GBIF, the geographic distribution of *M. flabellifolius* across the African continent is characterized by spatial unevenness and a certain degree of discontinuity. The most densely populated region was observed in the northeastern part of South Africa, with sporadic occurrences extending along Zimbabwe and Mozambique to Tanzania. The northernmost distribution extended to the central region of Kenya, albeit with a considerable geographical distance from areas in Tanzania. The westernmost distribution of *M. flabellifolius* on the African continent was observed in the western regions of Namibia and Angola, which are discontinuous areas in South Africa. In contrast, *M. moschatus* is primarily distributed in the central high-low regions of Madagascar (Figure 1).

### 2.2. Results of Niche Analysis

**Figure 2:**
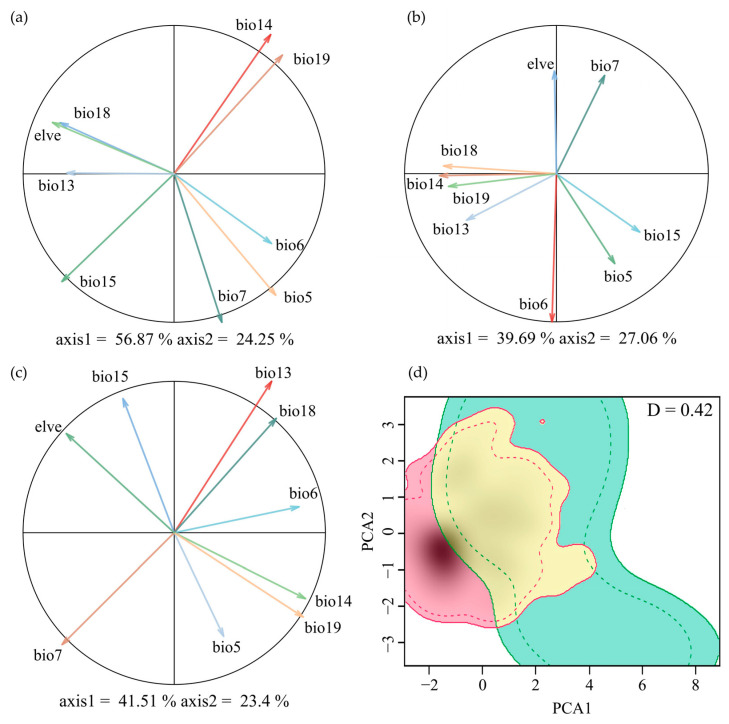
Ecological niche overlap between *M. flabellifolius* and *M. moschatus*. PCA plot generated from 9 environmental variables used to determine niche overlap dynamics of (**a**) *M. moschatus*, (**b**) *M. flabellifolius*, and (**c**) Myrothamnaceae; (**d**) the Schoener’s D Ecological niche overlap of the two Myrothamnaceae species, where green indicates *M. moschatus* and red is *M. flabellifolius*.

The dominant environmental factors contributing to the first principal component (PC1) differ between the two species. *M. moschatus* is associated with precipitation-related variables, while *M. flabellifolius* is associated with temperature-related variables. In the PCA analysis, the first two principal components (PCs) of *M. moschatus* explain 81.12% of the variation (56.87% and 24.25%, respectively), with the primary contributors to PC1 being Precipitation of the Driest Month (Bio14) and Precipitation of the Coldest Quarter (Bio19) and to PC2 being Max Temperature of the Warmest Month (Bio5) and Temperature Annual Range (Bio7) (Figure 2a). For *M. flabellifolius*, the first two principal components (PCs) explain 63.75% of the variation (39.69% and 27.06%, respectively), with the primary contributor to PC1 being Min Temperature of the Coldest Month (Bio6) and to PC2 being Precipitation of the Driest Month (Bio14) and Precipitation of the Warmest Quarter (Bio18) (Figure 2b). For Myrothamnaceae, the first two principal components (PCs) explain 64.91% of the variation (41.51% and 23.4%, respectively), with the primary contributors to PC1 being Precipitation of the Wettest Month (Bio13) and Temperature Annual Range (Bio7) and to PC2 being Precipitation of the Coldest Quarter (Bio19) and Precipitation of the Driest Month (Bio14) (Figure 2c).

**Figure A1:**
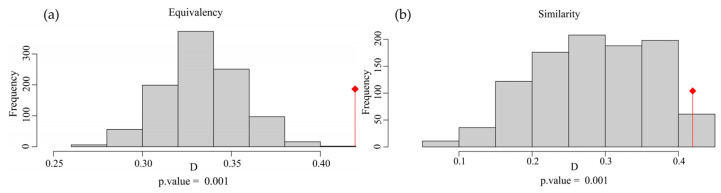
(**a**,**b**) Equivalency and similarity tests of Myrothamnaceae plants based on Schoener’s D. The red symbol represents the observed Schoener’s D.

The overall Schoener’s D for the combined factors of the two species was 0.420 (Figure 2d). In the test of niche equivalency, the observed Schoener’s D exceeds the upper limit of the 95% confidence interval obtained through random sampling, leading to the rejection of niche equivalency (Figure A1a). In the test of niche similarity, the observed Schoener’s D exceeds the 95% confidence interval upper limit from random sampling, supporting niche similarity (Figure A1b).

**Figure 3:**
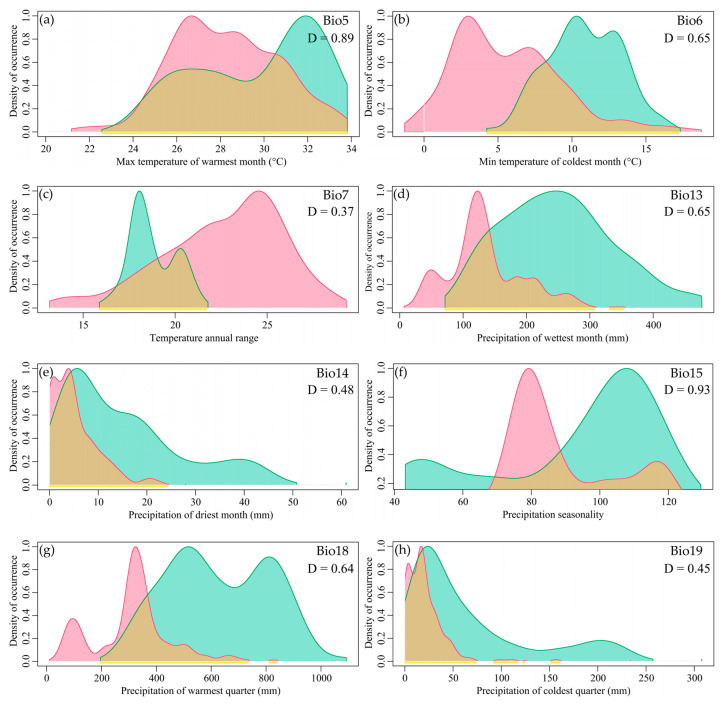
The niche spectrum of bioclimatic variables for the two species of Myrothamnaceae. The green patch represents *M. moschatus*, and red represents *M. flabellifolius*. (**a**) Max Temperature of the Warmest Month (Bio5); (**b**) Min Temperature of the Coldest Month (Bio6); (**c**) Temperature Annual Range (Bio7); (**d**) Precipitation of the Wettest Month (Bio13); (**e**) Precipitation of the Driest Month (Bio14); (**f**) Precipitation Seasonality (Bio15); (**g**) Precipitation of the Warmest Quarter (Bio18); (**h**) Precipitation of the Coldest Quarter (Bio19).

There was partial overlap in the niches of the two species within Myrothamnaceae, with most factors showing overlap exceeding 0.5. Looking at individual factors, the top two with the highest Schoener’s D were Precipitation Seasonality (Bio15) and Max Temperature of the Warmest Month (Bio5) (0.93 and 0.89), all exceeding 0.8 (Figure 3). The factors with the lowest Schoener’s D were Precipitation of the Driest Month (Bio14), Precipitation of the Coldest Quarter (Bio19), and Temperature Annual Range (Bio7) (0.48, 0.45, and 0.37), all below 0.5 (Figure 3).

The comparative analysis of the ecological factors between the two species revealed a significant shift in the optimal ranges of most ecological factors. The peak positions of Precipitation of the Driest Month (Bio14) and Precipitation of the Coldest Quarter (Bio19) for *M. moschatus* and *M. flabellifolius* were similar and located at lower levels, while the peak positions of other factors showed distinct separation. *M. flabellifolius* exhibited lower optimal values for the Max Temperature of the Warmest Month (Bio5) and Min Temperature of the Coldest Month (Bio6), whereas the optimal Temperature Annual Range (Bio7) was higher. Additionally, *M. flabellifolius* showed lower optimal values for Precipitation of the Wettest Month (Bio13) and Precipitation of the Warmest Quarter (Bio18), as well as smaller optimal values for Precipitation Seasonality (Bio15) (Figure 3a–h).

Regarding the ecological ranges of each factor, *M. flabellifolius* exhibited larger ecological ranges for Max Temperature of the Warmest Month (Bio5), Min Temperature of the Coldest Month (Bio6), and Temperature Annual Range (Bio7) compared to *M. moschatus*. However, for Precipitation of the Wettest Month (Bio13), Precipitation of the Driest Month (Bio14), Precipitation Seasonality (Bio15), Precipitation of the Warmest Quarter (Bio18), and Precipitation of the Coldest Quarter (Bio19), *M. moschatus* exhibited larger ecological ranges than *M. flabellifolius* (Figure 3a–h). This indicates that *M. flabellifolius* has a wider range of temperature adaptation, while *M. moschatus* has a broader range of adaptation to precipitation.

### 2.3. Evaluation of Species Distribution Model Results

**Figure 4:**
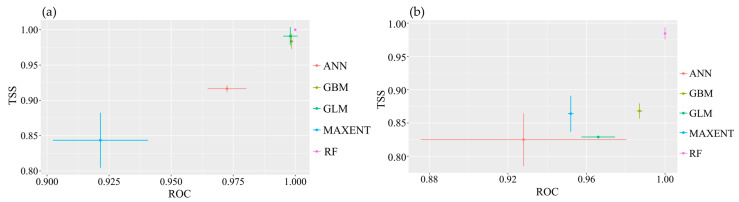
Evaluation test of single-model modeling for the two species of Myrothamnaceae. (**a**) *M. moschatus*; (**b**) *M. flabellifolius*.

**Figure A2:**
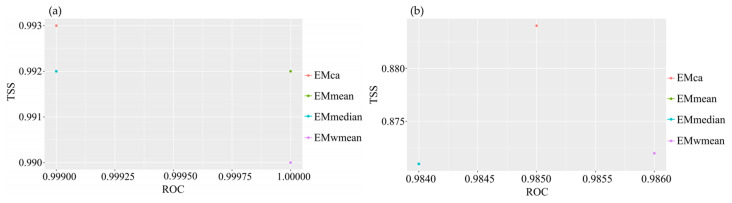
Evaluation results of ensemble modeling for two species of Myrothamnaceae. (**a**) *M. moschatus*; (**b**) *M. flabellifolius*. In (**b**), EMmean and EMwmean overlap.

**Figure A3:**
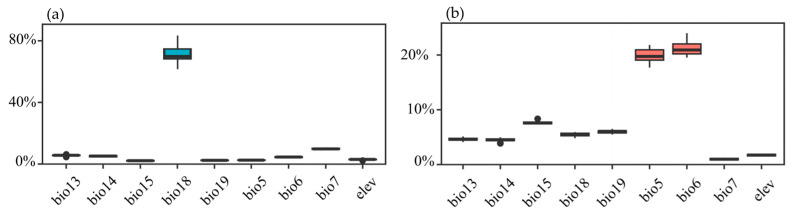
Contribution rates of each factor in the EMca ensemble model. (**a**) *M. moschatus*; (**b**) *M. flabellifolius*.

All single-model evaluation results for the two species of the Myrothamnaceae met the criteria of AUC > 0.9 and TSS > 0.8 (Figure 4), indicating excellent performance and predictive accuracy of the models. Across the four ensemble methods, namely, mean (EMmean), median (EMmedian), classification accuracy (EMca), and weighted mean (EMwmean), the AUC values were all above 0.9, and the TSS values were all above 0.8 for both species. We selected the classification accuracy (EMca) ensemble method with the highest TSS value (Figure A2). In the ensemble models, the highest contributing bioclimatic factors for the composite model of *M. flabellifolius* were the Max Temperature of the Warmest Month (Bio5) and Min Temperature of the Coldest Month (Bio6) (Figure A3b). For the composite model of *M. moschatus*, the highest contributing bioclimatic factor was the Precipitation of the Warmest Quarter (Bio18) (Figure A3a).

### 2.4. The Impact of Climate Change on the Suitable Habitat of Myrothamnaceae

**Figure 5:**
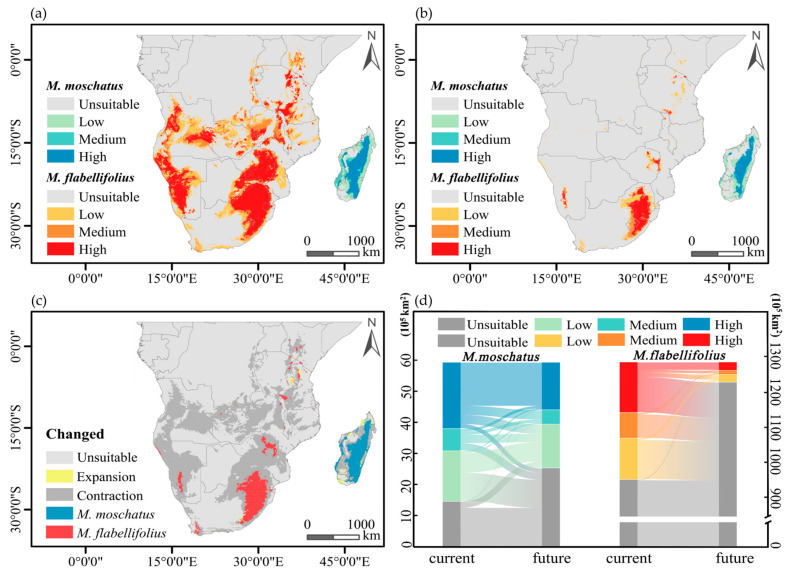
Current and future potential distribution patterns developed for *M. flabellifolius* and *M. moschatus*. (**a**) The potential distribution range of two Myrothamnaceae species currently; (**b**) the potential distribution range of two Myrothamnaceae species in the future (2100). (**c**,**d**) Demonstrates the predicted distributional area shift for either species from current to future.

The current suitable habitat of *M. flabellifolius* is primarily distributed on both sides of the African continent, south of the Sahara Desert. The high-suitability areas are mainly located in the northeastern edge of South Africa, eastern parts of Botswana, central and eastern regions of Zimbabwe, western regions of Namibia and Angola, and eastern regions of Angola, with sporadic distributions in the south and east of Zambia, Malawi, Mozambique, Tanzania, Kenya, Burundi, and the southern and eastern parts of the Congo (Figure 5a). The suitable habitat of *M. moschatus* in Madagascar is distributed in a belt-like pattern along the central-eastern part of the island, with high probability areas mainly located in the central-eastern region and sporadically in the western region (Figure 5a). Under climate change scenarios, the contiguous high-suitability areas of *M. flabellifolius* are only distributed on the northeastern edge of South Africa, with sporadic distributions in Zimbabwe, Malawi, Kenya, and Namibia (Figure 5b). The high-suitability areas of *M. moschatus* in Madagascar are distributed in a belt-like pattern along the central-eastern part of the island, with sporadic distributions in the western region (Figure 5b).

*M. flabellifolius* is projected to lose a large portion of its current suitable habitat under climate change, with only a small portion of new suitable habitat being added, mainly in Tanzania, Kenya, and South Africa (Figure 5c). The total suitable habitat of *M. flabellifolius* is estimated to be reduced by approximately 85%, with a proportionate loss of about 85% in high-suitability areas, where almost all medium- and low-suitability areas will become unsuitable regions. Among the lost high-suitability areas, about 83% will transition to unsuitable regions, 11% to low-suitability areas, and 6% to medium-suitability areas, while there will also be a 2% conversion from low-suitability areas and medium-suitability areas to high-suitability areas (Figure 5d).

*M. moschatus* is expected to lose some of its original suitable habitat, with new suitable habitats mainly distributed in the northwest and southwest of Madagascar, with a small amount in the eastern region (Figure 5c). The total suitable habitat of *M. moschatus* is projected to be reduced by approximately 29%, with a loss of about 34% in high-suitability areas and about 60% of medium- and low-suitability areas transitioning into unsuitable regions. Among the lost high-suitability areas, 18% will transition to unsuitable regions, 41% to low-suitability areas, and 41% to medium-suitability areas, while there will also be a 7% conversion from unsuitable regions, low-suitability areas, and medium-suitability areas to high-suitability areas (Figure 5d).

### 2.5. Variations in the Climatic Factors of the Natural Habitat of Myrothamnaceae

**Figure 6:**
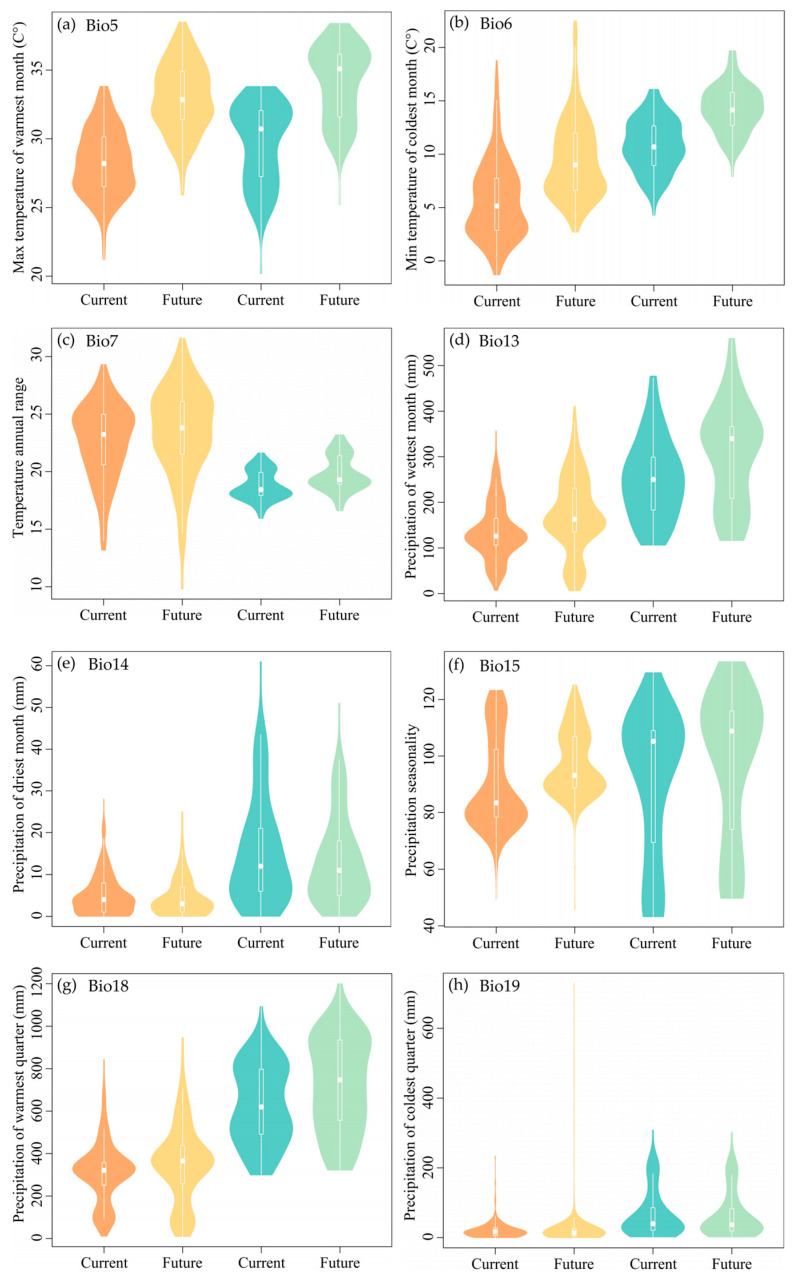
The pattern of the bioclimatic variables at the original habitats of Myrothamnaceae under current and future scenarios. Orange and yellow colors represent *M. flabellifolius* and the blue and green colors represent *M. moschatus*. (**a**) Max Temperature of the Warmest Month (Bio5); (**b**) Min Temperature of the Coldest Month (Bio6); (**c**) Temperature Annual Range (Bio7); (**d**) Precipitation of the Wettest Month (Bio13); (**e**) Precipitation of the Driest Month (Bio14); (**f**) Precipitation Seasonality (Bio15); (**g**) Precipitation of the Warmest Quarter (Bio18); (**h**) Precipitation of the Coldest Quarter (Bio19).

The values for Max Temperature of the Warmest Month (Bio5), Min Temperature of the Coldest Month (Bio6), and Precipitation Seasonality (Bio15) associated with the habitats of *M. flabellifolius* are projected to increase, showing considerable variation in the future. However, the Temperature Annual Range (Bio7), Precipitation of the Wettest Month (Bio13), and Precipitation of the Warmest Quarter (Bio18) are expected to exhibit an upward trend, but with smaller magnitudes of change. Precipitation of the Driest Month (Bio14) is predicted to show a slight decreasing trend with minimal variation, while Precipitation of the Coldest Quarter (Bio19) is expected to show no significant change (Figure 6a–h).

Projected changes indicate that the habitats of *M. moschatus* will experience an increase in the max Temperature of the Warmest Month (Bio5) and min Temperature of the Coldest Month (Bio6) with significant variation. However, Temperature Annual Range (Bio7), Precipitation of the Wettest Month (Bio13), Precipitation Seasonality (Bio15), and Precipitation of the Warmest Quarter (Bio18) are expected to also show an upward trend but with smaller magnitudes of change. Precipitation of the Driest Month (Bio14) is predicted to display a slight decreasing trend with minimal variation, while Precipitation of the Coldest Quarter (Bio19) is anticipated to show no significant change (Figure 6a–h).

## 3. Discussion

### 3.1. Niche Differentiation and Climate Adaptation of Myrothamnaceae

Analyses of niche overlap for two species of Myrothamnaceae, along with tests of equivalency and similarity, indicate that their ecological niches exhibit partial similarity. Myrothamnaceae exhibit similar adaptations to seasonal drought. The high overlap observed in Precipitation Seasonality (Bio15) and the similar peak positions at lower levels in Precipitation of the Driest Month (Bio14) and Precipitation of the Coldest Quarter (Bio19) suggest similar adaptations of the two species to precipitation seasonality, extreme dry month precipitation, and cold season precipitation. This adaptation may be attributed to their desiccation tolerance, allowing them to undergo dehydration dormancy to cope with seasonal drought during climatic aridity [16,17]. For the drought-tolerant species, Bio15 would be an important factor that contributes to their distribution. They predominantly grow in geographically harsh environments with exposed rock formations, where few other plant species can survive due to the severe climatic conditions, resulting in minimal or limited competition [24]. In more temperate climates, they may struggle to compete with taller vegetation, thus developing unique yet similar adaptations to precipitation factors. This finding supports the concept of niche conservatism, where closely related species, such as sister species, maintain similar ecological niche characteristics despite separate evolutionary paths [25,26]. Such conservatism likely reflects their shared evolutionary history and adaptations to similar environmental challenges.

While the ecological niches of *M. flabellifolius* and *M. moschatus* share similarities, there are also distinct differences observed in certain climatic niche factors. Some climatic niche factors show that the optimal ranges of these two species differ. The results suggest that *M. flabellifolius* may be better adapted to environments that are drier, with lower rainfall, lower temperatures, and greater annual temperature variations, compared to *M. moschatus*. Furthermore, the first principal component factors of the ecological niche differ: precipitation-related factors dominate the first principal component of the *M. moschatus* niche, while temperature-related factors dominate the first principal component of the *M. flabellifolius* niche. Additionally, *M. moschatus* displays a broader range of adaptation to precipitation, whereas *M. flabellifolius* shows a broader range of adaptation to temperature. This suggests that the preferences of the two species have diverged, likely due to long-term adaptive evolution resulting from their distinct geographical environments. *M. flabellifolius* and *M. moschatus* are allopatric species distributed on the African continent and the island of Madagascar [16,27]. Phylogenetic studies suggest that these two species diverged approximately 7 million years ago [18]. The distinct climatic conditions of Africa and Madagascar have led to differences in climate selection between these two species, likely due to their prolonged isolation and evolutionary histories [18]. Ecological selection occurs when new environmental conditions arise or geographical heterogeneity occurs, and this ecologically based divergent selection can create genetic diversity from the ancestral population, leading to speciation [28].

The differentiation of ecological niches is often closely related to morphological and structural differentiation [29,30]. Drought-resistant plants typically possess specific structures and characteristics that help reduce transpiration to cope with aridity, such as stomatal distribution and structure, leaf morphology, etc. [31,32]. The results indicate that *M. flabellifolius* may be adapted to a more arid niche compared to *M. moschatus*. However, the morphological structures of *M. flabellifolius* are more conducive to promoting transpiration than those of *M. moschatus*. The stomata of *M. flabellifolius* are not recessed, while those of *M. moschatus* are recessed; *M. flabellifolius* has hydathodes, while *M. moschatus* does not; and the leaf blades of *M. flabellifolius* are fan-shaped with fissures, while those of *M. moschatus* are lanceolate and unlobed [16,17]. The variation in leaf functional traits between *M. flabellifolius* and *M. moschatus* may be determined by their unique drought resistance strategy of desiccation tolerance. Transpiration serves as one of the primary driving forces for water movement within plants [33]. The structures in *M. flabellifolius* that promote transpiration facilitate rapid desiccation and dormancy induction when it encounters drought, enabling it to survive arid periods. Additionally, these structures may aid in rapid water absorption when precipitation occurs during desiccation. The presence of spiral-shaped conduits in *M. flabellifolius* that are absent in *M. moschatus* further supports this notion [16]. Conduits with spiral structures typically exhibit higher water transport efficiency, flexibility, and elasticity, making them more adaptable to mechanical stress and fluctuations [34]. In summary, this morphological explanation might help us in understanding why *M. flabellifolius* is more adapted to arid niches than *M. moschatus*.

Species of Myrothamnaceae demonstrate the complexity and diversity of ecological niches. Although they exhibit similar traits in adapting to harsh conditions such as extreme seasonal drought, these species have diverged in their ecological niches concerning temperature and precipitation factors. The main factors influencing the ecological niche of *M. flabellifolius* are related to temperature, while those of *M. moschatus* are related to precipitation. Moreover, *M. flabellifolius* is better adapted to ecological niches with less rainfall and lower temperatures compared to *M. moschatus*. The species niche differentiation not only reflects differences in their adaptation to climatic conditions but also correlates closely with geographical distribution and habitat differences. By considering the diverse ecological niche requirements and adaptive capacities of Myrothamnaceae plants, targeted conservation plans can be developed, such as implementing habitat restoration projects that cater specifically to their ecological needs.

### 3.2. Response of Myrothamnaceae to Climate Change

Climate is a fundamental determinant of species distribution, influencing various aspects, including range, life history traits, ecological niche, and adaptability [35]. Most regions of Africa are currently experiencing a trend of rising temperatures and decreasing rainfall, leading to an expansion of arid areas [22]. A previous study suggested that the expansion of arid regions may lead to the enlargement of habitats for drought-tolerant plants [21]. However, our predictions for Myrothamnaceae plants in Africa, which are known for their drought tolerance, suggest that they will all face threats of habitat loss and fragmentation under future climate change scenarios (SSP5-8.5, 2081–2100 years). Habitat loss and fragmentation can lead to population declines and population isolation, subsequently reducing genetic diversity and increasing survival pressure on species [35,36]. However, our study exclusively considers future climate change impacts, overlooking the pressures from human activities in Africa. Most African countries are expected to experience rapid population growth in the coming decades [37]. Population expansion will accelerate urbanization and industrialization, exerting pressure on the environment. Africa faces environmental challenges such as water scarcity, land desertification, deforestation, and ecosystem degradation [38,39]. Therefore, if no measures are taken, the threat to the survival space of Myrothamnaceae in the future will be greater than anticipated in our study. Our results indicate that the overall habitat for Myrothamnaceae is projected to decline in the future, although there are minor expansions in a few areas. Attention to these regions is crucial for their conservation.

Identifying different potential suitable areas during species distribution modeling is crucial for understanding the possibilities and changes in species distribution [40]. However, the transformation of suitable areas with different suitability levels under climate change has been overlooked in many studies on species distribution modeling. Our results indicate that under the influence of climate change, *M. flabellifolius* in Africa will experience a loss of approximately 85% of its total suitable habitat, with highly suitable areas losing about 85%. Of the lost highly suitable areas, 83% will be converted into unsuitable areas, while 17% will transition into moderate- to low-suitability areas, and the original moderate- to low-suitability areas will almost entirely change into unsuitable areas. Compared to *M. flabellifolius*, *M. moschatus* will currently lose about 29% of its total suitable area, with 34% of highly suitable areas lost. However, 18% of the lost highly suitable areas will be converted to unsuitable areas and 82% will be converted to moderate- to low-suitability areas, while the original moderate- to low-suitability areas also experience about a 60% reduction in suitability. Therefore, *M. moschatus* also faces significant risks of habitat loss. The transformation of suitable areas has important implications for the distribution of species and their ecological niches, as well as the internal structure and stability of ecosystems [41,42]. To address the changes in different suitability level areas, targeted conservation strategies are required, including the protection of highly suitable habitats, restoration of ecosystems in moderate- to low-suitability areas, and regulation of factors contributing to climate change.

Species have specific survival strategies, resource utilization patterns, and adaptability to environmental changes within their ecological niches, and these differences will influence their response to climate change [43]. Our results demonstrate varying degrees of reduction in suitable habitats for the two species of Myrothamnaceae in the future scenario, indicating different levels of adaptability to climate change. Specifically, *M. flabellifolius* experiences a much larger reduction in suitable habitat compared to *M. moschatus*. This disparity can be attributed to the primary influencing factor in the ecological niche of *M. flabellifolius*, which is the Min Temperature of the Coldest Month (Bio6); simultaneously, the highest contribution to the model is from the Max Temperature of the Warmest Month (Bio5) and Bio6. These temperature factors are expected to undergo considerable changes under future climate scenarios. In contrast, the main influencing factors in the ecological niche of *M. moschatus* are the Precipitation of the Driest Month (Bio14) and Precipitation of the Coldest Quarter (Bio19), as well as the highest contribution to the model, the Precipitation of the Warmest Quarter (Bio18). These precipitation factors are expected to experience minimal or no significant changes under future climate scenarios.

The impact of climate change on Myrothamnaceae species is substantial. The impact of climate change varies between these two species, possibly due to differences in adaptability stemming from niche differentiation and the uneven climate changes across different regions. They will face severe habitat loss, particularly *M. flabellifolius*. This study has solely focused on the impact of climate change on Myrothamnaceae, omitting consideration of factors such as human activities. Without effective intervention, the future threat to Myrothamnaceae may surpass our current projections. However, Myrothamnaceae species are not currently listed on the IUCN endangered species red list [23]. Each species is an irreplaceable part of Earth’s ecosystems, and their disappearance could disrupt the ecological balance, potentially triggering cascading effects that endanger human survival and development [44]. Therefore, it is imperative to take action to mitigate the impacts of climate change on Myrothamnaceae, enhance predictions of species responses to climate change, and promote effective conservation.

## 4. Materials and Methods

### 4.1. Species Occurrence and Environmental Data

The occurrence data for *M. flabellifolius* and *M. moschatus* were obtained from the Global Biodiversity Information Facility (GBIF, http://www.gbif.org, accessed on 8 October 2023) using the “rgbif” R package version 3.7-8 [45,46]. After removing records with errors, anomalies, and duplicates, we obtained 372 distribution records for *M. flabellifolius* and 108 distribution records for *M. moschatus*. Climate factor data and elevation data were downloaded from the WorldClim database version 2.1, with a resolution of 2.5 min. The climate factor data included 19 bioclimatic variables for both the current (1970–2000) and future (2081–2100) periods [47]. For future climate scenarios, predictions from the Beijing Climate Center Climate System (BCC-CSM2) under the highest carbon emission scenario (SSP5-8.5) were utilized [48]. To avoid multicollinearity caused by high correlations between factors, a Pearson correlation analysis was conducted on the 19 bioclimatic variables (Pearson r < 0.75) (Table A1) [49]. Finally, nine environmental factors were selected for subsequent analysis: Max Temperature of the Warmest Month (Bio5), Min Temperature of the Coldest Month (Bio6), Temperature Annual Range (Bio7), Precipitation of the Wettest Month (Bio13), Precipitation of the Driest Month (Bio14), Precipitation Seasonality (Bio15), Precipitation of the Warmest Quarter (Bio18), Precipitation of the Coldest Quarter (Bio19), and Elevation (Elev).

### 4.2. Niche Analysis

In this study, the “ecospat” R package version 3.5-1 was utilized for ecological niche analysis of species and to analyze the relative contributions of environmental variables to interspecific niche differentiation [50]. Initially, environmental data corresponding to the distribution points of the two species were extracted using the “raster” R package version 3.6-26 [51]. Subsequently, principal component analysis (PCA) of environmental factors was conducted separately for each species, as well as for the entire family, to calculate the relative contributions of each driving factor to interspecific niche differentiation. The ecological niche overlap was analyzed using Schoener’s D (D), with values ranging from 0 to 1, where 0 indicates no overlap and 1 indicates complete overlap [52].

Additionally, ecological niche equivalency and similarity tests were performed to assess the niche relationships between the two species. The niche equivalency test and niche similarity test utilize 1000 random permutations of environmental data to assess niche overlap between species. The equivalency test checks if niches are statistically equivalent by comparing the observed D value to a distribution generated from shuffled assignments of the data [53]. Conversely, the similarity test determines significant overlap by comparing the observed D value against a distribution from samples randomly drawn to match another species sample size [54]. If the observed D value for the equivalency test lies within the 95% confidence interval of the randomized distribution, or if it surpasses the 95% upper confidence limit in the similarity test, it indicates niche equivalence or significant overlap, respectively.

### 4.3. Model Construction and Distribution Simulation

For the construction of models for *M. flabellifolius* and *M. moschatus*, 400 and 100 pseudo-absence points were randomly generated, respectively, and integrated with species occurrence data. The number of pseudo-absence points was determined based on the actual number of distribution points. These points were then combined with current environmental factor data to build models. Model construction and distribution area simulation were performed using the “biomod2” R package version 4.2-4 [55]. Five single-model approaches, including Generalized Linear Models (GLMs), Generalized Boosted Models (GBMs), Artificial Neural Network Models (ANNs), Random Forest Models (RFs), and Maximum Entropy Models (MAXENTs), were employed for modeling. Seventy-five percent of the distribution data samples were randomly selected as training data, while the remaining twenty-five percent were used as testing data to evaluate model performance. This training–testing data split was repeated five times for each model. All models were evaluated using the Area under the ROC curve (AUC) and True Skill Statistic (TSS) [56].

Excellent models with AUC > 0.9 and TSS > 0.8 were selected, and four ensemble methods, including Ensemble Mean (EMmean), Ensemble Median (EMmedian), Ensemble Classification Accuracy (EMca), and Ensemble Weighted Mean (EMwmean), were used for combination to build ensemble models. The ensemble models were further evaluated using AUC and TSS, and the optimal ensemble model was selected for predicting potential suitable areas for the species under current and future climate scenarios. Based on the prediction results, suitable areas were classified into highly suitable (750–1000), moderately suitable (500–750), low suitable (250–500), and unsuitable (0–250) zones [57]. The current and future suitable area sizes were calculated in R version 4.2-3, and their dynamic changes were analyzed.

The figures related to geographic maps were plotted using ArcGIS 10.2, and the statistical graphs were drawn using R 4.2-3 [58].

## Data Availability

All data are available from the corresponding author upon reasonable request.

## Appendix A

**Table A1:** Pearson correlation analysis has been conducted on 19 climate factors and elevation.

Factors	Bio1	Bio2	Bio3	Bio4	Bio5	Bio6	Bio7	Bio8	Bio9	Bio10	Bio11	Bio12	Bio13	Bio14	Bio15	Bio16	Bio17	Bio18	Bio19	Elev
Bio1	1.00	−0.02	−0.14	0.08	0.81	0.65	0.08	0.80	0.84	0.89	0.80	−0.19	−0.10	−0.14	0.42	−0.15	−0.13	−0.36	0.06	−0.81
Bio2		1.00	−0.59	0.69	0.50	−0.69	0.90	0.28	−0.36	0.29	−0.43	−0.65	−0.53	−0.45	0.47	−0.52	−0.50	−0.57	−0.39	0.20
Bio3			1.00	−0.88	−0.60	0.55	−0.86	−0.49	0.24	−0.52	0.41	0.68	0.50	0.58	−0.50	0.49	0.64	0.55	0.44	0.12
Bio4				1.00	0.59	−0.63	0.92	0.57	−0.33	0.52	−0.53	−0.76	−0.70	−0.38	0.33	−0.69	−0.44	−0.54	−0.43	−0.07
Bio5					1.00	0.12	0.64	0.87	0.48	0.96	0.34	−0.58	−0.44	−0.38	0.57	−0.46	−0.41	−0.67	−0.17	−0.59
Bio6						1.00	−0.69	0.20	0.87	0.28	0.93	0.42	0.39	0.28	−0.09	0.36	0.33	0.18	0.42	−0.60
Bio7							1.00	0.48	−0.33	0.48	−0.48	−0.75	−0.62	−0.50	0.48	−0.61	−0.56	−0.63	−0.45	0.04
Bio8								1.00	0.44	0.93	0.32	−0.53	−0.48	−0.24	0.43	−0.51	−0.26	−0.46	−0.23	−0.66
Bio9									1.00	0.58	0.92	0.15	0.18	0.07	0.14	0.15	0.10	−0.13	0.32	−0.71
Bio10										1.00	0.45	−0.50	−0.39	−0.29	0.50	−0.43	−0.31	−0.57	−0.13	−0.72
Bio11											1.00	0.29	0.34	0.11	0.14	0.30	0.15	0.01	0.32	−0.64
Bio12												1.00	0.90	0.56	−0.52	0.92	0.62	0.76	0.62	0.07
Bio13													1.00	0.28	−0.21	0.99	0.33	0.63	0.53	0.03
Bio14														1.00	−0.60	0.30	0.98	0.52	0.39	−0.02
Bio15															1.00	−0.26	−0.64	−0.48	−0.37	−0.17
Bio16																1.00	0.34	0.65	0.55	0.06
Bio17																	1.00	0.56	0.43	−0.03
Bio18																		1.00	0.22	0.12
Bio19																			1.00	−0.09
Elev																				1.00

Note: Bio1: Annual mean temperature; Bio2: Mean diurnal range; Bio3: Isothermality; Bio4: Temperature seasonality; Bio5: Max temperature of the warmest month; Bio6: Min temperature of the coldest month; Bio7: Temperature annual range; Bio8: Mean temperature of the wettest quarter; Bio9: Mean temperature of the driest quarter; Bio10: Mean temperature of the warmest quarter; Bio11: Mean temperature of the coldest quarter; Bio12: Annual precipitation; Bio13: Precipitation of wettest month; Bio14: Precipitation of driest month; Bio15: Precipitation seasonality; Bio16: Precipitation of wettest quarter; Bio17: Precipitation of driest quarter; Bio18: Precipitation of warmest quarter; Bio19: Precipitation of coldest quarter; Elev: Elevation.
